# Commonalities Between ARDS, Pulmonary Fibrosis and COVID-19: The Potential of Autotaxin as a Therapeutic Target

**DOI:** 10.3389/fimmu.2021.687397

**Published:** 2021-10-04

**Authors:** Konstantinos Ntatsoulis, Theodoros Karampitsakos, Eliza Tsitoura, Elli-Anna Stylianaki, Alexios N. Matralis, Argyrios Tzouvelekis, Katerina Antoniou, Vassilis Aidinis

**Affiliations:** ^1^ Institute of Bio-Innovation, Biomedical Sciences Research Center Alexander Fleming, Athens, Greece; ^2^ Department of Respiratory Medicine, School of Medicine, University of Patras, Patras, Greece; ^3^ Laboratory of Molecular & Cellular Pneumonology, Department of Respiratory Medicine, School of Medicine, University of Crete, Heraklion, Greece

**Keywords:** COVID-19, ARDS, pulmonary fibrosis, Autotaxin, lysophosphatidic acid

## Abstract

Severe COVID-19 is characterized by acute respiratory distress syndrome (ARDS)-like hyperinflammation and endothelial dysfunction, that can lead to respiratory and multi organ failure and death. Interstitial lung diseases (ILD) and pulmonary fibrosis confer an increased risk for severe disease, while a subset of COVID-19-related ARDS surviving patients will develop a fibroproliferative response that can persist post hospitalization. Autotaxin (ATX) is a secreted lysophospholipase D, largely responsible for the extracellular production of lysophosphatidic acid (LPA), a pleiotropic signaling lysophospholipid with multiple effects in pulmonary and immune cells. In this review, we discuss the similarities of COVID-19, ARDS and ILDs, and suggest ATX as a possible pathologic link and a potential common therapeutic target.

## COVID-19

Severe acute respiratory syndrome coronavirus 2 (SARS-CoV-2) infection leads to the development of Coronavirus Disease 2019 (COVID-19), proclaimed pandemic on March 11, 2020 (1, 2). Upon airborne, mainly, CoV-2 transmission, the viral spike (S) glycoprotein mediates viral entry *via* binding to angiotensin-converting enzyme 2 (ACE2), supported by the transmembrane protease serine protease 2 (TMPRSS2) that proteolytically processes the S protein (3, 4). Infected cells in the lung, as detected with immunocytochemistry, include the upper airway bronchiolar epithelium and submucosal gland epithelium, as well as alveolar epithelial cells and macrophages (1). ACE2 is distributed mainly in the intestine, heart, kidney, as well as the lung, where alveolar epithelial type II cells are the major expressing cells. TMPRSS2 is highly expressed in several tissues; in the lung is co- expressed with ACE2 in nasal epithelial cells and alveolar epithelial type II cells, which might partially explain the tissue tropism of CoV-2 infection (3, 4).

CoV-2 infection is either asymptomatic or causes only mild respiratory diseases (non-pneumonia or mild pneumonia) in most individuals. However, a significant number of elderly individuals, frequently with comorbidities (such as cardiovascular diseases, diabetes, obesity), will develop a more severe form of the disease and will require hospital care (1, 2). COVID-19 most common clinical manifestations include fever, fatigue and dry cough, and dyspnea in severe cases (5). Severe COVID-19 associated histopathological changes are found mainly in the lungs, characterized by diffused alveolar damage (DAD), hyaline membranes and fibrin deposits, as well as severe endothelial injury, capillary microthrombi and exudative inflammation (6–12). A systematic review of published case reports and studies identified three main COVID-19 histological patterns: epithelial (85%), vascular (59%) and fibrotic (22%), with a frequent overlap (60%), whereas the epithelial and vascular patterns were present in all stages of severe COVID-19 (13).

## COVID-19, ARDS and Pulmonary Fibrosis

The rapid replication of SARS- CoV-2 and the associated epithelial cell death may, depending on the underlying genetic, inflammatory or metabolic context, trigger alveolar macrophages to produce excessive amounts of cytokines (such as TNF, IL-1b, IL-6, MIP1, IFN-γ and VEGF), a “cytokine storm”, associated with systemic infections such as sepsis or immunotherapies aftermath (14). The highly divert cytokine profile of COVID-19 hyperinflammation resembles, in some cases, other cytokine release syndromes, such as macrophage activation syndrome (15), although it is more heterogeneous and less robust, both quantitively (levels) and qualitatively (number of elevated cytokines). Noteworthy, IL-6 was found to be the most consistently upregulated cytokine and among the most overall predictive biomarkers (16, 17). In turn, the excessive production of cytokines further induces lung injury and Acute Respiratory Distress syndrome (ARDS), leading frequently to respiratory and multi organ failure and death (18).

ARDS develops most commonly in the setting of bacterial and viral pneumonias, or non-pulmonary sepsis, and is characterized by focal epithelial damage and excessive alveolocapillary permeability, leading to interstitial and alveolar edema and hypoxemia in the acute phase (18). Many severe COVID-19 patients will develop ARDS with impaired gas exchange and characteristic CT findings; however, the combination of multiple pathogenetic stimuli in COVID-19-induced ARDS results in a highly heterogeneous, “atypical” clinical appearance that has stimulated considerable controversy (2, 19–24). Nevertheless, excessive inflammation and endothelial dysfunction are among the top candidate pathologic events linking ARDS and COVID-19 (21, 25, 26) and markers of endothelial dysfunction have been recently correlated with COVID-19 mortality (27). Moreover, endothelial dysfunction is also a major characteristic of the most common comorbidities of COVID-19 that are associated with worse prognosis, hypertension, diabetes and obesity (21, 25).

The initial acute exudative inflammatory phase of ARDS is followed by a proliferative phase characterized by alveolar epithelial cell hyperplasia (18). A subset of acute ARDS survivors will further develop a fibroproliferative response, including fibroblast accumulation, deposition of collagen and other lung extracellular matrix (ECM) components (28), the magnitude of which was associated with ARDS duration (29). Moreover, and although invasive mechanical ventilation has revolutionized the management of ARDS, ventilator associated lung injury is considered as an additional contributor to pulmonary fibrosis in ventilated ARDS patients (30). Accordingly, a literature review of published histopathological analyses of COVID-19 lungs *postmortem* indicated, beyond DAD and hyaline membranes, the frequent presence of pulmonary fibrosis (31), while abnormal pulmonary architecture and functions have been reported in many recovering COVID-19 patients (32–34), suggesting persisting fibrotic abnormalities, pending large-scale and long-term follow up studies. Finally, CoV-2 infection *per se* has been reported to induce the expression of different pro-fibrotic factors including TGFβ (35). On the other hand, patients with interstitial lung diseases (ILD) had increased odds for ARDS development and severe COVID-19 (12, 36–39), while COVID-19-related acute exacerbation of ILDs had worse prognosis than non-COVID ILD acute exacerbations (40), thus suggesting pulmonary fibrosis both as a disease risk and a possible complication of COVID-19.

## Autotaxin (ATX; *ENPP2*) and COVID-19

ATX is a secreted glycoprotein that can be detected in most biological fluids, including blood and bronchoalveolar lavage fluid (BALF) (41). A large percentage (~40%) of serum ATX is thought to originate from the adipose tissue (42), while the damaged liver has been suggested as an additional possible source of serum ATX (43). High ATX expression has been reported from endothelial cells in high endothelial venules (HEVs) (44, 45), however their expected relative contribution to circulating levels should be low. Inflammatory macrophages have been also reported to express ATX upon inflammation (46–48), thus contributing to BALF ATX levels (46).

ATX is a constitutively active lysophospholipase D, that catalyzes the extracellular hydrolysis of lysophosphatidylcholine (LPC) to lysophosphatidic acid (LPA) (49). LPC is synthesized from fatty acids or membrane phosphatidylcholine (PC) by phospholipase A_2_ (PLA_2_) enzymes and is highly abundant in the plasma, associated with oxidized low-density lipoprotein (oxLDL) and albumin (50, 51). LPA is a growth factor-like signaling phospholipid with numerous effects in most cell types through its G-protein coupled receptors (LPAR1-6) (51–53). ATX has been suggested to bind to cell surface integrins (54–56), thus avoiding clearance, as well as localizing LPA production to its adjacent receptors, that exhibit widespread distribution and overlapping functions (51, 57).

Viral infections have been shown to increase systemic ATX levels, including HCV, HIV and HBV (43, 58, 59), while LPA has been also shown to directly affect HCV viral infection and replication (43, 60). Increased *ENPP2* mRNA expression was detected in nasopharyngeal swabs from COVID-19 patients, likely from immune cells (61), suggesting that ATX/LPA might stimulate viral infections, that could include SARS-CoV-2.

Increased serum/plasma ATX levels have been also reported in different diseases, including different forms of cancer, liver diseases, as well as respiratory diseases such as asthma and idiopathic pulmonary fibrosis (IPF) (**Table 1**) (41, 51). A variety of methods has been utilized, while reported levels exhibit remarkable heterogeneity, with no apparent consensus on healthy levels (**Table 1**). Increased ATX serum levels were recently reported in non-surviving ARDS patients, where ATX serum levels were shown to be an independent prognostic factor for 28 day mortality, outperforming the established SOFA/APACHE scores (62). Plasma ATX levels correlated with mortality also in a cohort of patients with severe sepsis (63), suggesting a role for ATX/LPA in systemic hyperinflammation. ATX serum levels in ARDS correlated with the increased IL-6/IL-8 serum levels (62), further supporting an interplay of ATX/LPA with inflammation, as previously suggested in breast cancer (64). ATX serum levels also correlated with the severity of lung injury (62), while increased ATX BALF levels upon endotoxin-induced acute lung injury (ALI) (65, 66), and ventilator-induced lung injury (VILI) in mice (67) have been reported. Moreover, ATX BALF levels in ARDS patients were positively associated with inflammatory and fibrotic mediators (IL-6, IL-8, TNF-α, MMP-7, fibronectin, OSM, and SPARC), suggesting that ATX may also have a role in the fibrotic component of ARDS (62). In line with the above, increased ATX levels have been detected in IPF patients and fibrotic animal models (46, 68), where results from genetic and pharmacologic studies have established a pro-fibrotic role for ATX (46, 69–72). Increased serum ATX levels were very recently detected also in COVID-19 patients hospitalized in the intensive care unit (ICU) as compared with less severe patients hospitalized in the clinic (61), thus adding ATX expression to the commonalities of COVID-19, ARDS and pulmonary fibrosis, and suggesting ATX as a possible pathologic link.

**Table 1:** Autotaxin serum levels in patients of different inflammatory diseases and cancer.

Disease classification	PMID	Disease	Samples (M/F/M+F)	ATX^1^	Method
Viral hepatitis	33102751	Chronic hepatitis C	28	1.1 ± 0.8	Two-site enzyme immunoassay
		Non-alcoholic steatohepatitis	19	1.4 ± 0.4 *	
		Alcoholic steatohepatitis	15	1.2 ± 0.4 *	
		*vs.*	*vs.*	*vs.*	
		Chronic hepatitis B	38	0.9 ± 0.3	
	21419756	Chronic Hepatitis C (histologically proven fibrosis)	74	2.40 ± 0.96	Two-site enzyme immunoassay
		Chronic Hepatitis C (FibroScan proven fibrosis)	134	2.20 ± 1.22	
	27981605	Chronic viral hepatitis	14	0.19 (0.13 - 0.35) ^*^	ELISA
			21	0.17 (0.04 - 0.13)	
		*vs.*	*vs.*	*vs.*	
		Healthy controls	8	0.13 (0.02 - 0.20)	
			12	0.18 (0.09 - 0.35)	
	28425454	Chronic hepatitis C	292	1.16 (0.85 - 1.68) *^, #^	Two-site enzyme immunoassay
			301	1.64 (1.19 - 2.20) ^*^	
			593	1.39 (1.01 - 1.99) ^*^	
		*vs.*	*vs.*	*vs.*	
		Healthy controls	80	0.76 ^#^	
			80	0.82	
			160	0.76	
	31933517	Liver cirrhosis (multiple aetiologies)	240	1.58 ± 0.68 ^#^	Two-site enzyme immunoassay
			160	1.99 ± 0.73	
		Chronic hepatitis B	33	1.36 ± 0.62 ^#, ~^	
			17	1.82 ± 0.5	
		Chronic hepatitis C	64	1.62 ± 0.67 ^#, $^	
			66	2.09 ± 0.71	
		Non viral hepatitis	143	1.49 ± 0.71 ^#^	
			77	1.96 ± 0.79	
	29114991	Chronic hepatitis B	62	1.10 (0.85-1.24)	Two-site enzyme immunoassay
			39	1.36 (1.23-1.64)	
			101	1.22 (0.95-1.42)	
Non-viral liver disorders	25062038	Liver cirrhosis	181	0.77 ± 0.41 ^*, #^	ELISA
			89	0.86 ± 0.43 ^*^	
			270	0.81 ± 0.42 ^*^	
		*vs.*	*vs.*	*vs.*	
		Healthy controls	35	0.18 ± 0.04 ^#^	
			50	0.35 ± 0.47	
			85	0.26 ± 0.40	
	29568204	Non-alcoholic fatty liver disease	186	0.86 ^*^	Two-site enzyme immunoassay
		*vs.*	*vs.*	*vs.*	
		Healthy controls	160	0.76	
	30905718	Liver cirrhosis	50	0.44 ± 0.22 ^*^	ELISA
		*vs.*	*vs.*	*vs.*	
		Healthy controls	20	0.19 ± 0.06	
	31144415	Non-alcoholic fatty liver disease	173	0.67 ± 0.21 ^#^	Two-site enzyme immunoassay
			134	0.97 ± 0.36	
			307	0.81 ± 0.32	
Bile duct disorders	31186435	Primary sclerosing cholangitis	193	6.3 ± 3.0 ^#, *^	Homovanillic acid assay
			59	8.6 ± 4.9 ^*^	
			252	6.8 ± 3.7	
		*vs.*	*vs.*	*vs.*	
		Healthy controls	57	2.5 ± 0.7 ^#^	
			142	3.2 ± 1.5	
	31651244	Primary biliary cholangitis – Severe	25	1.25 (0.72 - 4.31)	Two-site enzyme immunoassay
		*vs.*	*vs.*	*vs.*	
		Primary biliary cholangitis – Moderate	94	1.08 (0.58 - 3.12)	
	27506882	Primary biliary cholangitis	118	10.2 ± 4.4	Homovanillic acid assay
		Primary sclerosing cholangitis	115	7.3 ± 3.4	
		*vs.*	*vs.*	*vs.*	
		Healthy controls	Undisclosed	3.1 ± 1.7	
			Undisclosed	2.5 ± 0.7	
			109	2.8 ± 1.4	
	29802350	Primary biliary cholangitis	20	1.00 (0.82 - 1.13) *^,#^	Two-site enzyme immunoassay
			108	0.78 (0.66 - 0.98) ^*^	
			128	0.97 (0.79 - 1.11) ^*^	
		*vs.*	*vs.*	*vs.*	
		Healthy controls	80	0.76 ^#^	
			80	0.82	
			160	0.76	
	25450205	Preeclampsia / HELLP syndrome	17	16.8 ± 8.9	Homovanillic acid assay
		Pruritic disorders of pregnancy	33	16.8 ± 6.7	
		Intrahepatic cholestasis of pregnancy	55	43.5 ± 18.2 ^*, †^	
		*vs.*	*vs.*	*vs.*	
		Normal pregnancy	44	19.6 ± 5.4 ^*^	
		*vs.*	*vs.*	*vs.*	
		Healthy controls	57	2.5 ± 0.7 ^#^	
			142	3.2 ± 1.5	
Malignancies	2464234	Hepatocellular carcinoma	105	1.94 ± 1.01 ^#^	Two-site enzyme immunoassay
			43	2.87 ± 0.76	
			148	2.21 ± 1.03	
	18710386	Acute myeloid leukemia	26	0.86 ± 0.29	ELISA
		Chronic lymphocytic leukemia	14	0.93 ± 0.30 ^*^	
		Follicular lymphoma	25	1.47 ± 0.69 ^*^	
		Diffuse large B-cell lymphoma	28	0.94 ± 0.39 ^*^	
		*vs.*	*vs.*	*vs.*	
		Healthy controls	74	0.66 ± 0.12 ^#^	
			46	0.85 ± 0.18	
			120	0.73 ± 0.18	
	27583415	Hepatocellular carcinoma	58	1.07 (0.84 - 1.37) ^*^	Two-site enzyme immunoassay
		*vs.*	*vs.*	*vs.*	
		Healthy controls	74	0.68 ± 0.12 ^#^	
			46	0.97 ± 0.17	
			120	0.73 ± 0.18	
	29724718 ^a^	Non-small cell lung cancer	19	0.124 ^*^	TOOS assay
		*vs.*	*vs.*	*vs.*	
		Healthy controls	49	0.088	
	30921203	Breast cancer	112	0.29 ± 0.04 ^*^	ELISA
		*vs.*	*vs.*	*vs.*	
		Healthy controls	50	0.25 ± 0.02	
Metabolic disorders	26727116	Obese – overweight people >60 yo	20	0.17 ± 0.01 ^#^	ELISA
			40	0.29 ± 0.02	
			60	0.25 ± 0.11	
	26831013	Diabetic nephropathy	38	0.75 ± 0.27	ELISA
Autoimmune disorders	22493518 ^b^	Rheumatoid arthritis	10	0.87 ± 0.83 ^*^	ELISA
			16	1.12 ± 1.08 ^*^	
			26	1.03 ± 0.98 ^*^	
		*vs.*	*vs.*	*vs.*	
		Osteoarthritis	11	0.27 ± 0.19	
			15	0.32 ± 0.19	
			26	0.30 ± 0.19	
	24984830	Multiple sclerosis	20	12.11 ± 1.42 ^*^	TOOS assay
		*vs.*	*vs.*	*vs.*	
		Other neurological disorders	20	7.05 ± 1.51	
Various disorders	26083365	Chronic liver diseases	18	1.37 ± 0.77 ^*^	Two-site enzyme immunoassay
			17	1.46 ± 0.67 ^*^	
		Follicular lymphoma	10	0.95 ± 0.27 ^*^	
			15	1.28 ± 0.47 ^*^	
		*vs.*	*vs.*	*vs.*	
		Healthy controls	76	0.98 ± 0.58 ^#^	
			98	1.49 ± 0.98	
	32826822	Sepsis	84	443.6 (285.8 - 632.2)	TOOS assay
	33568105	Pancreatic diseases	114	0.39 ^*^	ELISA
		Benign pancreatic diseases	94	0.27	
		*vs.*	*vs.*	*vs.*	
		Healthy controls	120	0.26	
	34130757	Acute respiratory distress syndrome (survivors)	31	39.01 ± 13.89	Human Magnetic Luminex Assay
		Acute respiratory distress syndrome (non-survivors)	21	44.79 ± 13.38	

Only publications analyzing more than 10 samples are included.

^1^All reported values were converted to mg/L and presented as in the original publication as means ± SD, or as median (interquartile range). Individual values represent medians unless stated otherwise.

^*^: Compared to the same sex group of the controls; p < 0.05.

^#^: Compared to within-the-group opposite sex; p < 0.05.

^†^: Compared to females with normal pregnancy.

^~^: Compared to non-viral hepatitis.

^$^: Compared to hepatitis B.

a : ATX activity mean values are indicated.

b : ATX concentration in the serum was calculated anew by utilizing the supplementary data of this publication.

ATX levels in severe COVID-19 patients correlated with the increased IL-6 serum levels (61), as recently also shown in ARDS (62), as well in acute-on-chronic liver failure (ACLF) patients (73), suggesting interdependent regulation of expression. Accordingly, IL-6 has been reported to stimulate ATX expression from adipocytes (73) and human dermal fibroblasts (74). *Vice versa*, LPA has been reported to stimulate the expression of IL-6 from synovial fibroblasts (75, 76) and dermal fibroblasts (74), suggesting an ATX/LPA/IL-6 expression loop. Among the different components of the cytokine storm, IL-6 is the most predictive biomarker in COVID-19 (16, 17), correlating with respiratory failure and the need for mechanical ventilation (77), as well as with mortality risk (78).

Beyond hyperinflammation, and/or as its consequence, endothelial dysfunction is a major characteristic of COVID-19/ARDS (21, 25, 26). The increased ATX levels that were detected in severe COVID-19 patients correlated with markers of endothelial dysfunction (sP-sel, sICAM) (61) that have been independently correlated in the same samples with COVID-19 mortality (27). Similarly, ATX correlated with angiopoietin-2 levels and mortality in severe septic patients (63). In support for a major role of ATX/LPA on vascular homeostasis, ATX expression and LPA signaling have been shown necessary for the embryonic development of the vascular (and neural) system in mice (79–81). In adult mice, in studies unraveling the molecular mechanisms of SARS-CoV and MERS-CoV pathogenesis in the Collaborative Cross mice, *Enpp2*, the gene encoding ATX, has been reported to be a high priority candidate gene for pulmonary hemorrhage (82, 83). More importantly, LPAR1 null mice were reported to be protected from bleomycin (BLM)-induced pulmonary fibrosis, attributed to fibroblast accumulation and reduced vascular leak (68), as well as from *Candida albicans* water-soluble fraction (CAWS)-induced vasculitis, attributed to reduced CXCL1/IL-8-mediated neutrophil infiltration (84). Noteworthy, the stability of LPAR1 in the context of acute lung injury in mice has been proposed to be regulated by ubiquitination (84).

## LPA Signaling in Pulmonary and Immune Cells

Overall, any ATX effect will rely on its local levels (locally produced and/or extravasated) and its possible cell surface attachment, the local availability of LPC, the cell-specific expression profile of LPA receptors, as well as of the expression of the transmembrane lipid phosphate phosphatases (PLPP1-3; PPA2 A-C) that catabolize LPA (41, 85, 86). In this context, the possible effects of increased ATX levels can be deduced from the corresponding effects of LPA in the relative cells in the tissue microenvironment in question.

A plethora of LPA effects on pulmonary non-immune cells *in vitro* have been reported, as previously reviewed (87–89) and as summarized at **Table 2**. These include the promotion of apoptosis and the secretion of chemotactic signals (IL-8, MCP-1, CXCL1) from epithelial cells, the integrin-mediated activation of TGFβ on epithelial and smooth muscle cells, the modulation of permeability, leukocyte adhesion and cytokine secretion from endothelial cells, and the chemoattraction and accumulation of myofibroblasts (**Table 2**). LPAR1 has been reported as the main receptor mediating these effects, involving different well-known G-protein mediated pathways (**Table 2**). Moreover, LPA has been reported to transactivate different growth factors including TGFβ, PDGF and EGF that activate similar signal transduction pathways, while LPA was reported to signal also *via* RAGE (**Table 2**), further increasing the pleiotropic complexity of LPA signaling in the lung.

**Table 2:** Reported Lysophosphatidic acid (LPA) effects on pulmonary, non-immune, cells.

Cell type	LPA effect	Receptor		Pathway	PMID
**Epithelial cells**					
Human bronchial epithelial cells	induction of anchorage dependent apoptosis	LPAR1		-	22021336
	induction of TSLP & CCL20	–		-	18757306
	activation of TGF-β	LPAR2		integrin α_v_β_6_	19147812
	induction of soluble ST2 expression	LPAR1, 3		-	21871564
	transactivation of EGFR & secretion of IL-8	-		-	16687414 , 16197369
	induction of IL-13Ra2	–		G_αi_	17287216
	enhancement of epithelial barrier integrity	LPAR1, 3		-	19586906 , 17359381
	decrease of EGFR-EGF binding	–		-	17640953
	induction of COX-2 expression & PGE2 secretion			G_αi_	18294142
	transactivation of PDGFR-β	–		–	12890682
	redistribution of c-Met on the membrane	-		-	17689924 , 23624790
Human bronchial epithelial cells (BEAS-2B)	transactivation of EGFR	LPAR1	–		17640953
	inhibition of IFN/TNF-induced CCL5/RANTES expression	LPAR1	G_i_/PI3K		20861350
	decrease of EGFR-EGF binding	–	–		17640953
Human alveolar epithelial carcinoma cells (A549)	decrease of p53 abundance	-	-		18025263
	increase of cell migration	LPARs	PKCδ, cortactin		21696367
	promotion of EMT, proliferation and migration	RAGE	PKB		33109194
Human basal cells	induced signaling by CREB	–	ERK1/2		33794877
Mouse alveolar and bronchial epithelial cells	induction of apoptosis	LPAR1	-		22021336
	induction of apoptosis	LPAR2	–		23808384
Mouse lung epithelial cells (MLE12)	induction of migration	LPAR1	TrkA		26597701
	induction of KC secretion	LPAR1	ERK, p38		27448760
**Endothelial cells**					
Human microvascular endothelial cells	increase of the endothelial layer permeability	LPAR2, 6	-		23084965
Human pulmonary artery endothelial cels	increased adhesive properties	LPAR1, 3	–		25621161
Human aortic endothelial cells	induction of VCAM, E-selectin	-	G_i_		10595650
	induction of E-selectin, MCP-1, monocytic migration and adhesion	–	ROCK2, NF-κB		30884801
	induction of VCAM, ICAM	-	ROCK2, NF-κB		20164172
Human endothelial cells	CXCL1 secretion, monocyte adhesion	LPAR1, 3	–		21531341
Human airway epithelial cells	inhibition of the attachment to the ECM	LPAR1	Rho-kinase		27500235
Mouse endothelial cells	vascular leak/extravasation	LPAR1	–		18066075
Bovine pulmonary artery endothelial cells	migration, chemotaxis	-	ERK, Hic-5		17337598 15333043
		-	-		
**Fibroblasts**					
Human fibroblasts	chemoattraction, accumulation	LPAR1	–		18066075
	proliferation, EGFR ectodomain shedding	-	ERK1/2		21362091
	differentiation, profibrotic gene expression (TGFβ, col1a2, FN, SMA)	LPAR2	ERK1/2, Smad 3, Akt, p38		23808384
Mouse lung fibroblasts	lamelipodia formation, motility	LPAR1	-		14744855
Mouse fibroblasts (NIH 3T3)	migration, protection from apoptosis, proliferation	–	–		16219296
	protection from apoptosis, proliferation		G_i_		11062066
Rat Rat1/c-Myc fibroblasts	protection from apoptosis	–	Rac1		11062066
Mesenchymal cells derived from fibrotic lung allografts	promotion of NFAT1 nuclear translocation	LPAR1	β-catenin		28240604
**Smooth muscle cells**					
Human smooth muscle cells	proliferation, stimulation of EGFR signaling	-	-		9252534 , 11741820
	activation of TGF-β	–	integrin α_v_β_5_		22025551
Rabbit smooth muscle cells	contraction	-	-		9338431
**Stem cells**					
Human mesenchymal stem cells	migration	LPAR1	β-catenin		22782863
	migration, differentiation into myofibroblasts	LPAR1	–		24251962

The effects of ATX and LPA signaling on the regulation of immune cells have been previously reviewed in detail (87–92). Briefly, high ATX expression from ECs in HEVs in lymph nodes has been reported (44, 45), where ATX has been suggested to facilitate lymphocyte homing *via* the promotion of the adhesion (44), transmigration and motility of lymphocytes (45, 93, 94). Intriguingly, LPA signaling has been proposed to intersect with sphingosine phosphate (S1P) signaling (95), a closely related phospholipid (96), that has been shown to affect lymphocyte egress from the lymph nodes (97).

Non-withstanding the effects of ATX/LPA on lymphocyte homeostasis, highly pertinent for both ILD/IPF and COVID-19, a role for ATX/LPA on the homeostasis of the monocyte phagocyte system is emerging. Macrophages are central players in the pathogenesis of both IPF (98, 99) and COVID-19 (15, 100, 101), exhibiting remarkable heterogeneity and spatiotemporal plasticity. LPA has been suggested to stimulate the expression of macrophage chemotactic factors from ECs, such as monocyte chemoattractant protein-1 (MCP-1) (102) and CXCL1 (103), thus promoting both monocyte migration as well as adhesion to ECs (102–104). Beyond LPA-induced macrophage chemoattraction and adhesion to ECs, inflammatory macrophages *per se* have been reported to express ATX upon BLM-induced pulmonary inflammation and fibrosis, while IPF macrophages have been shown to stain for ATX (46). scRNAseq analysis of BALF cells from COVID-19 patients indicated a predominance of macrophages (100, 101), where *ENPP2* mRNA expression was detected in monocyte-derived alveolar macrophages (Mo-AMs) (61), that have been shown to drive the development of BLM-induced pulmonary fibrosis in mice (105). In turn, accumulating evidence indicates that LPA co-stimulate macrophage maturation and/or activation (47, 106–109), suggesting an autocrine role of ATX/LPA in macrophage pathologic responses. Moreover, LPA has been suggested to stimulate oxLDL uptake and foam cell formation (110, 111), linking macrophages and ATX/LPA with hyperlipidaemia and cardiovascular diseases (112), major comorbidities of COVID-19.

While LPA promotes bone marrow derived monocyte (CD11b^+^) activation (F4/80 expression) *in vitro* as potently as M-CSF (106), LPA has been also reported to co-stimulate the GM-CSF/IL-4-induced conversion of monocytes to DCs (113, 114). Moreover, LPA has been also reported to modulate the activity of TCF4 (115), a decisive transcription factor in plasmacytoid dendritic cells (pDCs) development and homeostasis (116). Increased *ENPP2* expression was detected in COVID-19 DCs and pDCs, correlating with markers of immature DCs (61), while an anti-inflammatory role of LPA has been previously proposed for DCs *via* LPAR2 (117), suggesting that ATX/LPA could be also involved in suppression of DC responses in COVID-19.

Therefore, increases of ATX levels and LPA local production in ARDS, ILD/IPF and COVID-19 can exacerbate numerous pathogenic responses in the lung, likely in co-ordination with other pathologic inflammatory and fibrotic factors.

## Pharmacologic Targeting of ATX as an Additional Therapeutic Option in COVID-19

Dexamethasone (Dex), the first line of defense against systemic inflammation, has been proven effective in COVID-19 patients requiring oxygen or ventilation (118, 119), the only approved single therapy against severe COVID-19. Remarkably, Dex treatment of ventilated COVID-19 patients attenuated serum ATX levels, suggesting that the therapeutic effects of Dex include the suppression of ATX expression (61) and that ATX can be druggable.

The exacerbated production of IL-6 and other storm cytokines, where present, is considered among the leading causes of COVID-19/ARDS-related mortality, and therefore many clinical trials have been conducted targeting storm cytokines or their receptors, with inconsistent results, spurring controversial opinions on the use of systemic anti-inflammatory drugs (120). ATX and IL-6 levels were shown to correlate in ACLF (73), ARDS (62) and COVID-19 (61) patients, suggesting that simultaneous inhibition of both IL-6 and ATX may be an effective therapeutic strategy for COVID-19, as previously suggested in systemic sclerosis (74).

**Figure 1:**
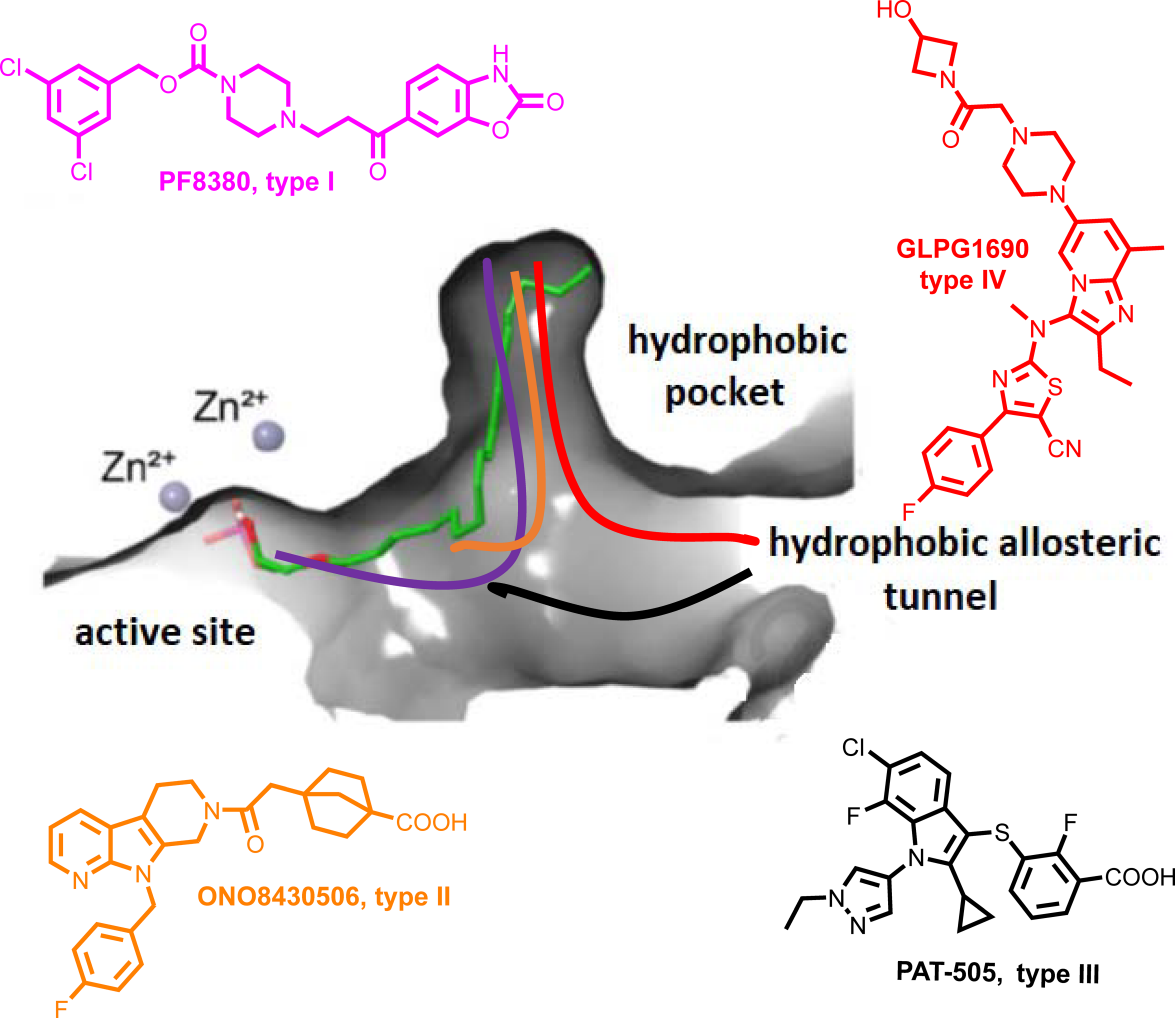
Schematic representation of the mode of binding (color coded) to ATX by the 4 different types of ATX inhibitors (I-IV). The mode of binding of LPA is also displayed (in green).

The antifibrotic compounds pirfenidone and nintedanib, approved for IPF, have shown efficacy in fibrotic lung diseases other than IPF (121–125). Therefore, since COVID-19 and IPF share disease severity risk factors, such as sex/age and comorbidities, existing and developing anti-fibrotic compounds have been suggested as additional therapeutic options in COVID-19 (126–128). Among them, GLPG1690 (**Table 3** and **Figure 1**) targets ATX and, together with the standard of care treatment (pirfenidone or nintedanib), has entered phase III international clinical trials (ISABELA 1 and 2, NCT03711162 and NCT03733444) (129). Given the above, the same or a similar regime might also prove effective in COVID-19.

**Table 3:** Representative small molecules targeting ATX in late-stage preclinical and clinical development.

Compound(Company)	*In vitro* properties			Pre-clinical data			Clinical trials
	IC50(assay)	Mode of Binding (Ki)ATX inhibitor type(PDB structure entry)	ADMET	Pharmacokinetics	LPA inhibition	Disease targeting(Dose, route)	
**GLPG-1690** **Ziritaxestat** **(Galapagos)**	131 ± 12 nM (hATX, TOOS assay)^1^ 418 nM (mouse plasma, 18:2 LPA, LC-MS/MS)^1^ 542 nM (rat plasma, 18:2 LPA, LC-MS/MS)^1^ 242 nM (human plasma, 18:2 LPA, LC-MS/MS)^1^	Competitive (15 nM, hATX), type IV inhibitor1 ( 5MHP )	hERG IC_50_: = 15 μM^1^ CYP3A4 TDI: negative^1^	**iv clearance (L/h·kg)**: 0.23 (mouse), 0.69 (rat), 0.12 (dog)^1^ **C_max_** (mouse, 30 mg/kg per os): 21.367 μg/mL^1^ **t_max_** (mouse, 30 mg/kg per os): 1 h^1^ **t_1/2_** (mouse, 30 mg/kg per os): 3.8 h^1^ per os bioavailability (F%): 29 (mouse), 37 (rat), 63 (dog)^1^	95% (maximum) (30 mg/kg per os)^1^	**Pulmonary fibrosis** (3, 10 or 30mg/kg per os)^1^	Phase I NCT03143712 NCT02179502 IPF Phase II NCT02738801 Phase III NCT03711162 NCT03733444 Scleroderma Phase II NCT03798366 NCT03976648
**BLD-0409** **Cudetaxestat** **(Blade Therapeutics)**	≤ 0.5 μM (LPC assay)^2^	–	–	–	–	**Metabolic disorders** (15mg/kg)^2^	Phase I NCT04146805 NCT04814472 NCT04814498 NCT04939467
**ONO-8430506** **(Ono Pharmaceuticals)**	5.1 nM (recomb. ATX, FS-3 assay)^3^ 10.2 nM (hATX, LPC assay)^3, 4^ 6.4 nM (mouse plasma, LPC assay)^3^ 19 nM (rat plasma, LPC assay)^3^ 5.5 nM (human plasma)^3, 4^	type II inhibitor	Protein plasma binding: rat (95.1%), human (99%)^4^ High selectivity to ATX^3^	**iv clearance (mL min-1 kg-1):** 8.2 (mouse), 4.7 (rat), 5.8 (dog)^4^ **Vdss (L/kg)**: 1.5 (mouse), 1.9 (rat), 2.3 (dog)^4^ **Cmax (1 mg/kg per os)**: 124 ng/mL (mouse), 261 ng/mL (rat), 1670 ng/mL (dog)^4^ **t1/2** (1 mg/kg per os): 5.4 h (mouse), 2.5 h (rat), 5.9 h (dog)^4^ **per os Bioavailability (F)**: 51.6% (rat), 71.1% (dog), 30.8% (monkey)^4^	96% (18:2 LPA, 3 mg/kg)^3^ 93% (20:4 LPA, 3 mg/kg)^3^ >99% (18:2 & 20:4 LPA, 30 mg/kg)^3^	**Prostatic hyperplasia** (0.3-10mg/kg, id)^3^ **Breast cancer** (10mg/kg, per os)^5^ **Thyroid cancer** (2mg/kg, per os)^6^	Preclinical evaluation
**PF-8380** **(Pfizer)**	2.8 nM (hATX, FS-3 substrate)^7^ 1.7 nM (hATX, LPC substrate)^7^ 1.16 nM (mATX, FS-3 substrate)^7^ 1.15 nM (fetal fibroblast cell line, LPC substrate)^7^ 101 nM (human whole blood)	Competitive (0.02-0.04 nM), type I inhibitor^7^	Solubility (pH = 6.8) = 0.011 mg/mL^8^ Poor solubility at physiological pH (7.4)^8^ IC50 hERG (cardiotoxicity) = 2.7 μM^8^ Permeability (PAMPA assay) = 81%^8^	**rat iv clearance (mL min-1 kg-1)** = 31^7^ **Vdss (L/kg)** = 3.2^7^ t1/2 = 1.2 h^7^ **Cmax** (10 mg/kg per os) = 2.55 μM^7^ **tmax** (10 mg/kg per os) = 0.67 h^7^ **rat per os F** (10 mg/kg) = 83%^7^	EC50 = 54.7 nM (16:0 LPA)^7^ EC50 = 84.6 nM (18:0 LPA)^7^ EC50 = 51.7 nM (20:0 LPA)^7^	**Arthritis Hyperalgesia** (30mg/kg, po)^7^ **Glioblastoma** (10mg/kg, ip)^9^ **Liver fibrosis** (30mg/kg, ip)^10^ **Lung allograft fibrosis** (30mg/kg, per os)^11^	Preclinical evaluation

^1-11^ refer to the following hyperlinked publications (PubMed ID): **1**: 28414242 , **2**: 33342311 , **3**: 24747415 , **4**: 32551021 , **5**: 24599971 , **6**: 25398768 , **7**: 20392816 , **8**: 29798825 , **9**: 24062988 , **10**: 27981605 , **11**: 28240604 .

The crystal structure of ATX has been solved (55, 130, 131), allowing a deep understanding of its structure and function relationship (132) (**Figure 1**), and thus promoting rational drug design. Given the establishment of ATX as a therapeutic target in IPF, as well as the promising results from the initial clinical trials, a plethora of ATX inhibitors have been developed (133, 134); the ones at late-stage development as shown in **Table 3** and their mode in binding at **Figure 1**.

Inducible genetic deletion of ATX in adult life, resulting in 70-80% decreases in serum ATX levels and mRNA expression levels in different tissues, did not have any appreciable effects in gross pathophysiology of major organs (135), suggesting that the bulk of ATX activity in mice is dispensable for adult life. Moreover, potent (IC50 2 nM), long term (3 weeks) pharmacological inhibition of ATX with PF-8380 (120 mg/Kg - 4 times the effective concentration; PO; bid) had no effects in survival or gross pathology of major organs (135), suggesting that ATX pharmaceutical targeting is safe and well tolerated in mice. In humans, GLPG1690 was reported to be well tolerated in a phase 1 randomized clinical trial (NCT02179502), safe and efficacious in a phase 2a randomized placebo-controlled clinical trial (NCT02738801), supporting ATX inhibition as a novel treatment for IPF (136, 137). In addition, administration of BBT-877, another orally available small molecule inhibitor targeting ATX (IC50 ~6.7 nM), to healthy volunteers in a phase I clinical trial (NCT03830125), did not reveal severe adverse events (138, 139). However, the GLPG1690 phase III clinical trial was recently discontinued on account of “low benefit to risk ratio“. Likewise, BBT-877’s scheduled phase II clinical trial was also postponed due to “toxicity concerns“. Since the relative results are not announced yet, it is not known if the toxicity was imposed from the compounds themselves or their target. Nevertheless, several new candidates are emerging, while possible compound toxicity can be eliminated with targeted modifications or bypassed *via* inhaled administration.

## Conclusions

The increased levels of ATX in ILDs/IPF, ARDS and COVID-19 add yet another commonality between them and suggest that LPA signaling is involved in their pathogenesis, including the amplification of vascular damage, the regulation of the immune system and the promotion of fibrosis. Therefore, the therapeutic targeting of ATX in IPF and fibrotic diseases could be also applied in COVID-19, alone or together with approved anti-fibrotic, anti-rheumatic and anti-viral drugs, especially given its predicted short-term administration, as well as the emergency nature and unmet medical need for the treatment of COVID-19 severe cases.

## Author Contributions

KN, TK, ET, ES and AM drafted the paper. AT and KA critically commented on the draft version. VA finalized the manuscript. All authors contributed to the article and approved the submitted version.

## Funding

This work has been co-financed by the European Union and Greek national funds through the Operational Program Competitiveness, Entrepreneurship and Innovation, under the call Research – Create – Innovate (project code: T1EDK-0049. The funders had no role in study design, data collection and analysis, decision to publish, or preparation of the manuscript.

## Conflict of Interest

The authors declare that the research was conducted in the absence of any commercial or financial relationships that could be construed as a potential conflict of interest.

## Publisher’s Note

All claims expressed in this article are solely those of the authors and do not necessarily represent those of their affiliated organizations, or those of the publisher, the editors and the reviewers. Any product that may be evaluated in this article, or claim that may be made by its manufacturer, is not guaranteed or endorsed by the publisher.
